# The role of long intergenic noncoding RNA 00511 in malignant tumors: a meta-analysis, database validation and review

**DOI:** 10.1080/21655979.2020.1795384

**Published:** 2020-07-25

**Authors:** Jianlong Ding, Junyan Cao, Zhaocong Chen, Zhiming He

**Affiliations:** a Department of Hepatobiliary Surgery, 3201 Hospital of Xi’an Jiaotong University Health Science Center, Shanxi Xi’an, China; b Department of Medical Ultrasonic, The Third Affiliated Hospital of Sun Yat-sen University, Guangzhou, Guangdong, China; c Department of Rehabilitation Medicine, The Third Affiliated Hospital of Sun Yat-sen University, Guangzhou, Guangdong, China; d General Surgery, Chongqing Red Cross Hospital, Jialing No.1 Village, Jiangbei District, Chongqing, China

**Keywords:** LINC00511, tumor, prognosis, metastasis

## Abstract

Increasing studies suggested that long intergenic noncoding RNA 00511 (LINC00511) could facilitate the progression of various malignancies and correlates with prognosis of patients with malignant tumors. However, its clinical significance is still not completely clarified. Therefore, we performed a meta-analysis and bioinformatics analysis to further evaluate the correlation of LINC00511 expression level with prognosis and metastasis in patients with tumors. The pooled hazard ratio (HR) with 95% confidence interval (CI) was used to evaluate the prognostic significance of LINC00511 expression level. The pooled odds ratio (OR) with 95% CI was applied to assess the association between LINC00511 expression level and tumor metastasis. A total of 12 studies involving 1040 tumor patients were included in this meta-analysis. The pooled analyses suggested that higher LINC00511 expression level correlated with worse overall survival (OS) (HR = 1.93, 95% CI 1.49–2.49, P < 0.001) and higher incidence of lymph node metastasis (OR = 3.07, 95% CI 2.23–4.23, P < 0.001). Additionally, bioinformatics analysis based on TCGA datasets also showed that increased LINC00511 expression level may predict poor OS and disease-free survival (DFS) in patients with malignant tumors. Taken together, our finding suggested that high LINC00511 expression level may be correlated with poor prognosis and high incidence of metastasis. Nevertheless, further large-scale and high-quality studies are needed to validate our findings.

## Graphical Abstract

**Figure:**
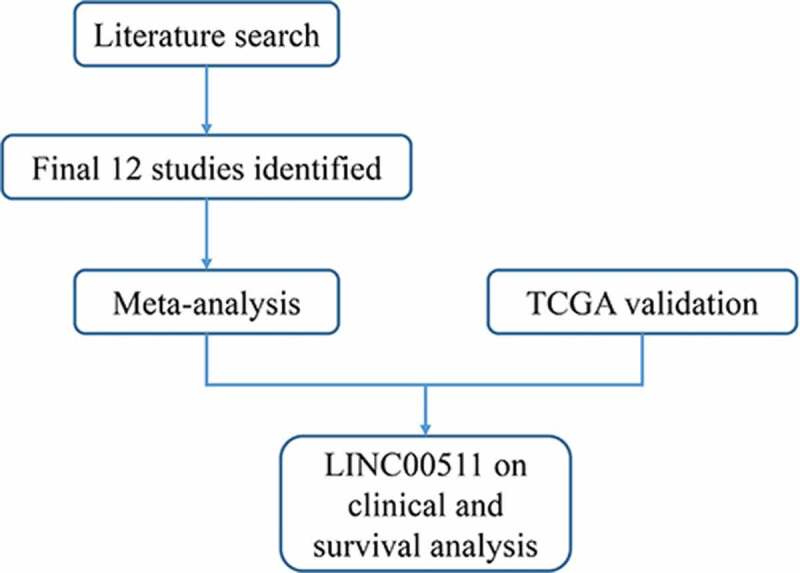

## Background

Cancer is a major cause of death and an essential public health problem worldwide [1]. According to GLOBOCAN 2018, there were approximately 18.1 million new cancer cases and 9.6 million cancer-associated deaths globally in 2018 [2]. Although enormous progress in diagnoses and treatments has improved survival, the long-term outcome of patients with advanced cancer is still unfavorable [3]. Therefore, it remains necessary and urgent to explore novel therapeutic targets and prognostic biomarkers for cancers [4].

Long noncoding RNAs (lncRNAs) are non-protein-coding single-stranded RNAs with longer than 200 nucleotides, which play crucial roles in multiple physiological processes, such as transcriptional regulation, posttranscriptional processing and decoy of miRNAs, chromatin remodeling and protein degradation [5]. Additionally, mounting evidence shows that many lncRNAs are dysregulated in tumorigenesis and could be exploited as novel therapeutic targets and prognostic biomarkers for cancers [6]. Long intergenic noncoding RNA 00511 (LINC00511) is a newly identified lncRNA and has been reported to be dysregulated in various malignant tumors, including glioma [7], ovarian cancer [8], breast cancer [9,10], cervical cancer [11,12], osteosarcoma [13], lung cancer [14], hepatocellular carcinoma [15–17], renal cell cancer [18], pancreatic cancer [19], gastric cancer [20–22], tongue squamous cell carcinoma [23] and papillary thyroid carcinoma [24]. To date, most literature suggested that LINC00511 was aberrantly upregulated in malignant tumors and its high expression correlated with poor survival of patients with glioma [7], ovarian cancer [8], breast cancer [9,10], cervical cancer [11,12], osteosarcoma [13], lung cancer [14], gastric cancer [22], hepatocellular carcinoma [15], renal cell cancer [18] or pancreatic cancer [19]. Furthermore, those studies revealed that LINC00511 could accelerate tumor progression by inhibiting malignant cell apoptosis and promoting tumor cell proliferation, migration, invasion, metastasis, chemotherapy resistance, and so on [10,11,17].

However, the association between LINC00511 expression and prognosis of patients with malignant tumor is still controversial. For example, Qiao et al. [13] reported that LINC00511 was downregulated in osteosarcoma, which was related to improved survival. Additionally, they further demonstrated that upregulation of LINC00511 could impede the progression of osteosarcoma through inducing apoptosis and suppressing the migration and invasion of osteosarcoma cells [13]. Moreover, the conclusions concerning the clinical role of LINC00511 expression in patients with malignancies in individual studies may be limited by small sample size. Therefore, in this study, we further evaluated the clinical significance of LINC00511 expression in malignant tumors through performing a meta-analysis of the current literatures and conducting a bioinformatics analysis using The Cancer Genome Atlas (TCGA) data. Meanwhile, we also made a comprehensive review of the mechanisms underlying the biological functions of LINC00511 in malignant tumors.

## Materials and methods

### Search strategy and study selection

We searched PubMed, EMBASE, Web of science, Wanfang and China National Knowledge Infrastructure (CNKI) databases from inception up to July 2020 for eligible studies. The search terms included ‘Long intergenic noncoding RNA 00511’ or ‘LINC00511’. The studies according with the following criteria simultaneously were included: 1) patients were divided into two groups based on LINC00511 expression level; 2) Hazard ratios (HRs) with 95% confidence intervals (CIs) for the association between LINC00511 expression and overall survival (OS) were available; and 3) studies were published in English or Chinese. The exclusion criteria were as following: 1) studies were letters, conference abstracts, meta-analysis or review articles; 2) studies evaluated the prognostic value of LINC00511 expression level using The Cancer Genome Atlas (TCGA) data; or 3) studies enrolled the overlapped patients.

### Data extraction and quality assessment

Two authors independently extracted data from eligible studies. Any disagreement was removed by discussion among all the authors. The extracted information covered first author, publication date, country, tumor type, treatment type, sample size, detection method, reference controls, cutoff values, HRs with 95% CIs for OS, survival analysis type and follow-up duration. When HRs were generated using both univariate and multivariate, the HRs from multivariate analysis were preferentially selected due to less confounding factors. If not directly provided in paper, HRs with 95% CIs would be calculated from Kaplan-Meier survival curves using Engauge Digitizer software. The methodological quality of studies was evaluated using Newcastle-Ottawa scale (NOS) criteria, which is a ‘star’ rating system from 0 to 9 stars [25].

### Validation by analyzing public data

This study conformed to the publication guidelines established by The Cancer Genome Atlas (TCGA). Gene Expression Profiling Interactive Analysis (GEPIA) was applied to assess the correlations of LINC00511 expression level with OS and DFS. The survival analysis was calculated using the K-M method and log-rank test, and the HRs and p value were presented in the figures of K-M curve as previously described [4].

### Statistical analyses

All data analyses were conducted using Stata software version 12.0 (Stata Corporation, College Station, TX, USA). HRs with 95% CIs were used to assess the correlation of LINC00511 expression level with OS or DFS. X^2^-based Cochran Q test and Higgins I^2^ statistic were applied to evaluate the heterogeneity among the eligible studies. P-value < 0.05 or I^2^-value > 50% indicated the presence of substantial heterogeneity [25]. The random-effect model was chosen when substantial heterogeneity existed. Sensitivity analyses were performed to explore the source heterogeneity and the robustness of the pooled HR for OS. Publication bias was examined by Begg’s funnel plots and Egger’s linear regression tests [25].

## Results

### Study selection and characteristics

**Figure 1.**
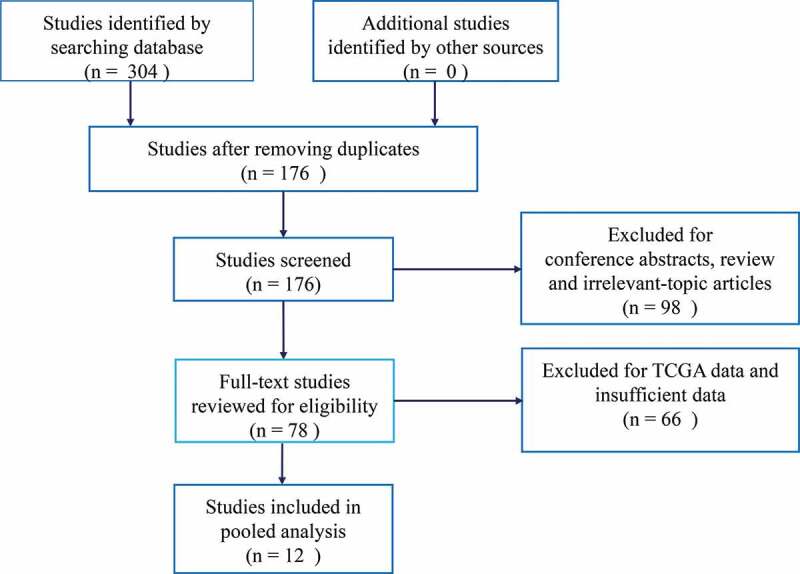
Flow diagram of the study selection.

A total of 304 potentially relevant articles were retrieved from electronic databases. To yield eligible articles, we firstly excluded 128 duplicate articles. After screening the titles and abstracts, we further excluded 98 articles for conference abstracts, review articles and irrelevant-topic studies. Then, 78 articles were screened by full-text. During this step, 66 studies were removed due to insufficient data and TCGA data. Finally, a total of 12 eligible studies were included in this meta-analysis [7–15,18,19]. The flow diagram summarizing the study selection procedure is presented in Figure 1. The main characteristics of eligible studies are summarized in Table 1. All studies enrolled Chinese patients and were published between 2016 and 2020. The sample sizes of eligible studies ranged from 39 to 140. Ten types of cancer were analyzed in eligible including hepatocellular carcinoma (n = 1), osteosarcoma (n = 1), breast cancer (n = 2), lung cancer (n = 1), glioma (n = 1), ovarian cancer (n = 1), cervical cancer (n = 2), renal cell carcinoma (n = 1), pancreatic cancer (n = 1) and gastric cancer (n = 1). LINC00511 expression levels were detected by RT-qPCR in all the eligible studies. All the included studies were given with no less than six scores based on NOS criteria, suggesting that they were moderate to high quality and eligible for this meta-analysis.

**Table 1.** The main characteristics of studies included in this meta-analysis.

Study	Tumor type	Country	Sample size	Detection method	Reference controls	Cutoff value	HR (95% CI) for OS	Multivariate analysis	Follow-up (months)	Treatment	NOS score	Reference
Deng. HH 2019	RCC	China	49	RT-qPCR	GAPDH	median	1.40 (1.02–1.91)	No	NA	Surgery	7	[18]
Huang HG 2020	GC	China	80	RT-qPCR	GAPDH	mean	1.41 (1.06–1.87)	No	NA	Surgery	7	[22]
Liu. L 2019	BC	China	98	RT-qPCR	GAPDH	median	1.54 (1.02–2.33)	No	NA	Surgery+ Radiotherapy	7	[9]
Lu. GM 2018	BC	China	39	RT-qPCR	GAPDH	mean	1.55 (1.12–2.14)	No	NA	Surgery	6	[10]
Mao. BD 2019	CC	China	84	RT-qPCR	GAPDH	median	1.46 (1.01–2.11)	No	60	Surgery	7	[11]
Qiao. SC 2019	Osteosarcoma	China	45	RT-qPCR	β-actin	median	0.038 (0.005–0.293)	Yes	NA	Surgery+ Chemotherapy	6	[13]
Sun. CC 2016	NSCLC	China	124	RT-qPCR	GAPDH	P25	7.19 (3.53–10.56)	Yes	NA	Surgery	7	[14]
Wang. J 2019	OC	China	80	RT-qPCR	GAPDH	NA	1.69 (3.53–10.56)	No	37	Surgery	7	[8]
Wang. B 2019	Glioma	China	82	RT-qPCR	GAPDH	mean	2.531 (1.532–4.183)	Yes	NA	Non	7	[7]
Wang. RP 2019	HCC	China	127	RT-qPCR	GAPDH	median	3.016 (1.216–3.889)	Yes	NA	Surgery	7	[15]
Yu. CL 2019	CC	China	92	RT-qPCR	GAPDH	median	2.895 (1.446–5.797)	Yes	NA	Surgery	7	[12]
Zhao. XH 2018	PDAC	China	140	RT-qPCR	GAPDH	NA	2.258 (1.989–2.563)	Yes	NA	Surgery	7	[19]

**Abbreviation**: HR: Hazard ratios; OS: overall survival; RCC: Renal cell carcinoma; BC: Breast cancer; CC: Cervical cancer; NSCLC: Non-small cell lung cancer; OC: Ovarian cancer; HCC: Hepatocellular carcinoma; PDAC: Pancreatic ductal adenocarcinoma;

### Meta-analysis and bioinformatics analysis of association between LINC00511 expression and survival outcomes

**Figure 2.**
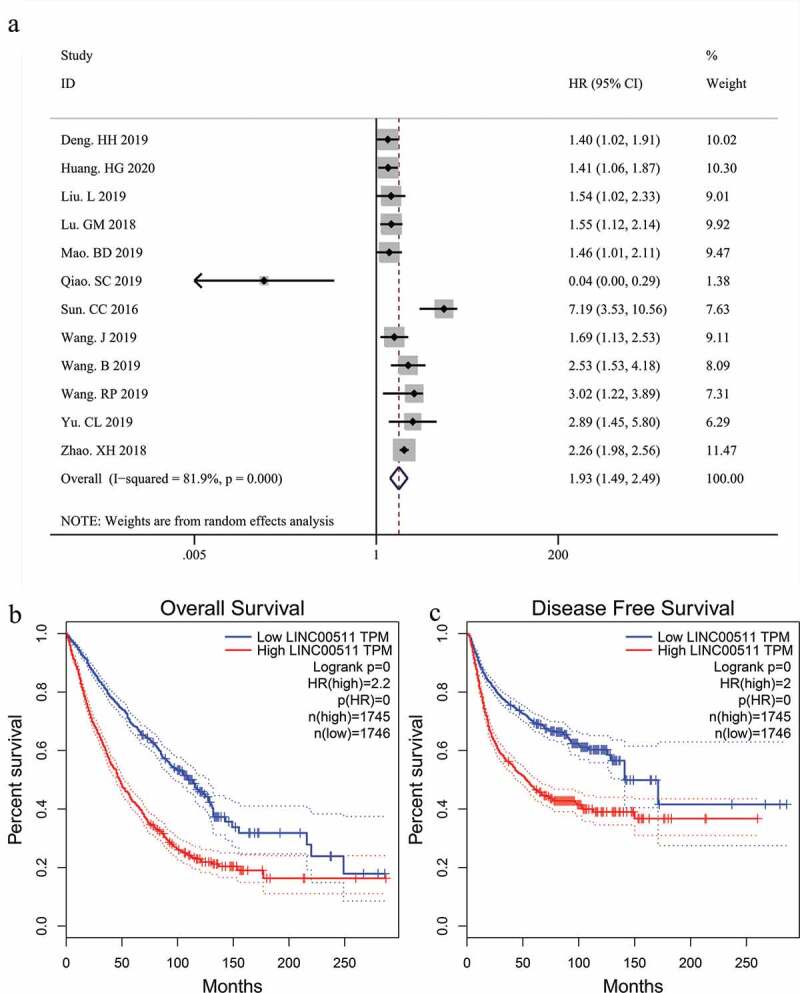
Forest plots of the association between LINC00511 expression level and overall survival in patients with malignant tumors(a); Kaplan-Meier survival analysis of LINC00511 expression for overall survival (b) and disease-free survival (c).

A total of 12 studies, which enrolled 1040 patients with malignant tumors, were included in our meta-analysis to assess the correlation between LINC00511 expression level and OS. Considering the obvious heterogeneity among these studies (I^2^ = 81.9%, p < 0.01), we applied the random-effect model to calculate the pooled HR and 95% CI for OS. The result showed that higher LINC00511 expression correlated with worse OS (HR = 1.93, 95% CI 1.49–2.49, P < 0.001) (Figure 2(a)). To date, only two studies reported the relationship between LINC00511 expression and progression-free survival and none of the studies referred to DFS, so we could not conduct a meta-analysis of the prognostic significance of LINC00511 expression for these survival outcomes. To confirm the result of our meta-analysis and evaluate the prognostic significance of LINC00511 expression for the other survival outcomes, we adopted survival plots in GEPIA through merging LINC00511 expression data and survival data of malignancies from all the TCGA dataset, in which 3491 patients were divided into high or low expression group according to LINC00511 expression. The results suggested that higher LINC00511 expression predicted worse OS and DFS (Figure 2(b–c)), further validating the result of our meta-analysis.

### Meta-analysis of association between LINC00511 expression and lymph node metastasis

**Figure 3.**
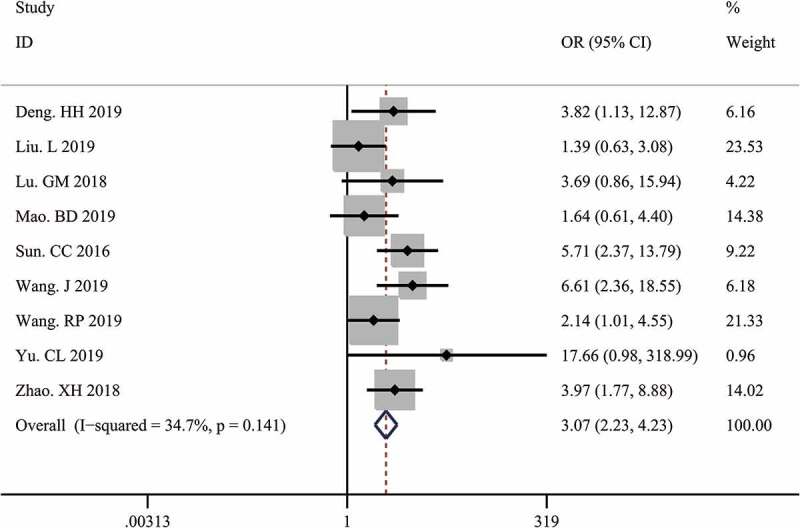
Forest plots of the association between LINC00511 expression level and lymph node metastasis.

A total of 9 studies with 833 tumor patients were included in this meta-analysis to assess the association between LINC00511 expression and lymph node metastasis. Considering the absence of significant heterogeneity among these studies (I^2^ = 34.7%, p = 0.141), we applied the fixed-effect model to calculate the pooled OR and 95% CI for lymph node metastasis. The result showed that higher LINC00511 expression correlated with higher incidence of lymph node metastasis (OR = 3.07, 95% CI 2.23–4.23, P < 0.001) (Figure 3). To date, only two studies reported the relationship between LINC00511 expression and distant metastasis, so we did not conduct a meta-analysis of the association between LINC00511 expression and distant metastasis due to small sample size.

### Sensitivity analysis and publication bias

**Figure 4.**
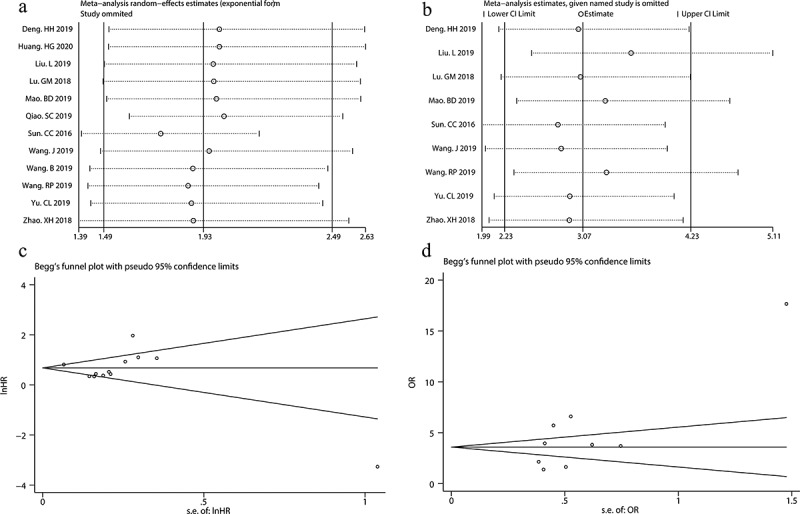
Sensitivity analysis of overall survival (a) and metastasis (b); Begg’s funnel plots of OS (c) and lymph node metastasis (d).

Sensitivity analysis was performed by sequentially omitting single study. As shown in Figure 4(a–b), removal of any individual study had no substantial effects on the pooled HR for OS and the pooled OR for lymph node metastasis. Begg’s funnel plot and Egger’s test were conducted to evaluate the publication bias. The results showed that Begg’s funnel plots for OS and lymph node metastasis were symmetric (Figure 4(c–d)). Moreover, the p values of Egger’s tests for OS and lymph node metastasis were 0.509 and 0.052, respectively. These results suggested that there was no significant publication bias in our meta-analysis. Overall, our sensitivity analysis and publication bias assessment indicated that our pooled estimations were robust and reliable.

## Discussion

Numerous studies have suggested that LINC00511 was dysregulated in a multiple of tumors and this correlated with prognosis and metastasis in human malignancies. However, the conclusions regarding the clinical significance of LINC00511 expression level in malignant tumors remain conflicting, so we performed a meta-analysis and bioinformatics analysis based on The Cancer Genome Atlas (TCGA) data to systematically assess the clinical significance of LINC00511 expression level. Additionally, we also made a comprehensive review of the biological functions of LINC00511 in malignant tumors and the relevant mechanisms. In this study, we found that increased LINC00511 expression was closely associated with worse OS and DFS, as well as higher incidence of lymph node metastasis. Taken together, these findings suggested that LINC00511 might serve as a biomarker for prognosis and metastasis in malignant tumors.

**Figure 5.**
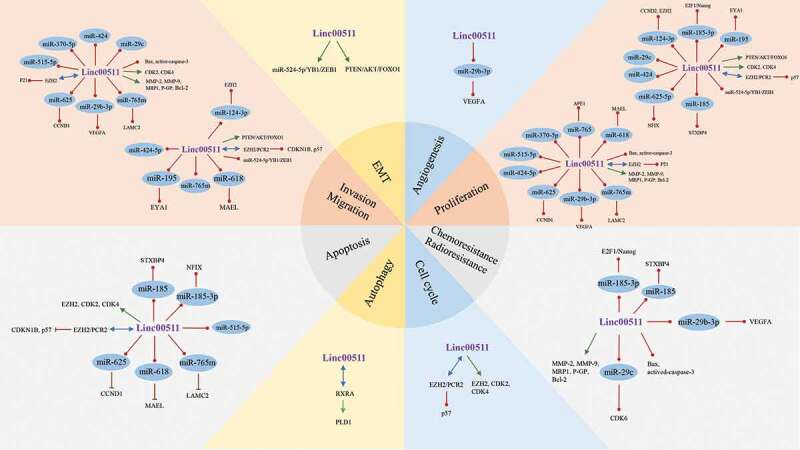
Biological functions of LINC00511 and relevant molecular mechanisms in malignant tumors.

The mechanisms underlying the carcinogenic roles of LINC00511 are rather complex (Table 2 & Figure 5), which may help to explain the clinical significance of LINC00511 expression level in tumors. First, most of studies revealed that LINC00511 could promote the aggressive phenotypes of malignant cells through sponging miRNAs and modulating the expressions of their targets, including miR-625/CCND1 [20], miR-515-5p [21], miR-185-3p/E2 F1/Nanog [10], miR-185/STXBP4 [9], miR-124-3p/CCND2 [26], miR-124-3p/EZH2 [22], miR-524-5p/YB1/ZEB1 [27], miR-195/EYA1 [17], miR-424 [15], miR-29 c [28], miR-29b-3p/VEGFA [19], miR-424-5p [29], miR-625-5p/NFIX [20], miR-370-5p [29], miR-765 m/LAMC2 [23], miR-618/MAEL [30], miR-29/CDK6 [28] and miR-765/APE1 [31] pathways. Second, LINC00511 can regulate tumor suppressors or oncogenes by interacting with DNA methylation-related enzymes and transcriptional factors to facilitate tumor progression. For example, in ER-negative breast cancer, LINC00511 could interact with enhancer of zeste homolog 2 (EZH2) and recruited polycomb repressive complex 2 (PRC2) to methylate lysine 27 on histone H3, which downregulated CDKN1B expression, ultimately facilitating cell cycle progression [32]. It was suggested that LINC00511 could promote the proliferation, invasiveness, metastasis but decreased apoptosis of lung cancer cells by interacting EZH2 to suppress p57 expression via the methylation of lysine 27 on histone H3 of p57 gene [14,33]. Additionally, LINC00511 was also demonstrated to downregulate the expressions of KLF2 and LATS2 by promoting the binding of LSD1 to the promoter regions of LATS2 and KLF2 in lung cancer cells [33]. In thyroid carcinoma, LINC00511 could interact with TAF1 to upregulate JAK2, which then activated STAT3 signaling pathways to maintain the resistance to radiotherapy of malignant cells [34]. In ovarian carcinoma, LINC00511 was able to suppress P21 expression by interacting with EZH2, subsequently inhibiting tumor cell proliferation [35]. Third, LINC00511 can accelerate tumor progression through regulating several tumor-associated signaling pathways such as RXRA/PLD1 [36], PTEN/AKT/FOXO1 [37], p38 [21], ERK1/2 [21] and JNK [21] pathways. Moreover, several studies suggested that LINC00511 could modulate the expressions of multiple proteins related to the aggressive phenotypes of tumor cells [11,24]. Nevertheless, how LINC00511 regulates these tumor-associated signaling pathways and proteins has not been unveiled, which deserves further explorations. Although the vast majority of literature supports the notion that LINC00511 may function as an oncogene, the opposite results were reported in osteosarcoma. Yan et al. [31] and Guo et al. [30] reported that LINC00511 acted as an oncogene in the progression of osteosarcoma. On contrast, Qiao et al. [13] found that high LINC00511 expression correlated with favorable prognosis, and it could impede osteosarcoma progression both in vitro and in vivo. Therefore, further studies are needed to further ascertain the biological functions of LINC00511 in osteosarcoma and the other malignant tumors.

**Table 2.** Clinical significance and biological functions of LINC00511 and the relevant molecular mechanisms in malignant tumors.

Tumor types	Tumor cell lines	Expression in tumor tissues and cell lines	Clinical significance of dysregulation	Related cell processes	Direct or indirect targets	References
Renal cell Cancer	A498, 786-O, ACHN and Caki-2	Increased	Advanced tumor stage, higher incidence of lymph node metastasis; Worse OS.	Facilitate proliferation, colony formation, cell cycle progression, and metastasis, and repress apoptosis in vitro; Accelerate tumor growth in vivo.	miR-625/CCND1	[18]
Gastric cancer	AGS, SGC7901, BGC823, MKN45 and MGC803	Increased	Advanced tumor stage, Larger tumor size; Worse OS and DFS.	Facilitate tumor cell proliferation, migration and invasion, and repress apoptosis in vitro; Accelerate tumor growth in vivo.	miR-515-5p; miR-625-5p/NFIX p38, ERK1/2 and JNK, miR-124-3p/EZH2	[20–22]
Breast cancer	MDA-MB-231, MDA-MB-436, MDA-MB-468, MDA-MB-453, and MCF-7	Increased	Higher incidence of lymph node metastasis, distant metastasis and recurrence, larger tumor size, and advanced tumor stage; Worse OS.	Facilitate proliferation, cell cycle progression, sphere-formation ability, invasion, resistance to radio therapy and repress apoptosis in vitro; Accelerate tumor growth in vivo and promote resistance to radio therapy in vivo.	miR-185-3p/E2F1/Nanog, miR-185-3p/STXBP4, EZH2/PRC2/CDKN1B	[9,10,28,32]
Glioma	U87, LN229, U251 and A172	Increased	Higher WHO grade and KPS score; Worse OS and DFS.	Facilitate proliferation, migration, invasion and EMT in vitro; Accelerate tumor growth in vivo.	miR-124-3p/CCND2 miR-524-5p/YB1/ZEB1,	[26,27]
Hepatocellular carcinoma	SMCC7721, HepG2,Hep3B MHCC-97 H, Huh7, HCC-LM3 and MHCC-97 L	Increased	Advanced tumor stage, higher incidence of lymph node metastasis and vascular invasion; Worse OS.	Facilitate proliferation, cell cycle progression colony formation and invasion and repress apoptosis in vitro.	miR-195/EYA1, miR-424, miR-29 c	[15–17]
Pancreatic ductal adenocarcinoma	PANC-1, MIA PaCa-2, Capan-2, SW1990, ASPC-1 and BxPC-3	Increased	Higher incidence of lymph node metastasis and early recurrence; Worse OS.	Facilitate proliferation, migration, invasion and angiogenesis.	miR-29b-3p/VEGFA	[19]
Non-small-cell lung cancer	A549, SK-MES-1, H1299, 95D, H460, H520, H1975, H157, SK-LU-1, and SPC-A-1	Increased	Large tumor size, Advanced tumor stage, and higher incidence of lymph node metastasis; Worse OS.	Facilitate proliferation, invasion metastasis, and EMT, and repress apoptosis in vitro; Accelerate tumor growth in vivo.	EZH2/PRC2/p57, PTEN/AKT/FOXO1, EZH2/KLF2, EZH2/LATS2, LSD1/LATS2, LSD1//KLF2	[14,33,37]
Thyroid cancer	B-CPAP, KTC-1, TPC-1, BCPAP and IHH-4	Increased	Large tumor size and higher incidence of lymph node metastasis	Facilitate proliferation, cell cycle progression migration, invasion, and repress apoptosis and radiosensitivity in vitro.	CDK2, CDK4, EZH2, TAF1/JAK2/STAT3	[24,34]
Ovarian cancer	CAOV3, OVCAR3 SKOV3, and UWB1.289	Increased	Large tumor size, Advanced tumor stage, and higher incidence of lymph, node metastasis, poor differentiation; Worse OS.	Enhance cell viability and invasion, and decrease apoptosis in vitro	miR-424-5p, miR-370-5p, EZH2/P21	[29,35]
Cervical cancer	SiHa, HeLa, C33A and Caski	Increased	Large tumor size, Advanced tumor stage, and higher incidence of lymph, node metastasis, distant metastasis and squamous cell carcinoma; Worse OS.	Facilitate proliferation, cell cycle progression, migration, invasion, and resistance to paclitaxel, and repress apoptosis and autophagy	RXRA/PLD1,MMP-2, MMP-9, P-GP, Bcl-2, MRP1, Bax and active caspase-3	[11,12,36]
Tongue squamous cell carcinoma	Tca-8113, SCC-9 SCC-4 and CAL-27	Increased	Not been studied	Facilitate proliferation and invasion in vitro	miR-765 m/LAMC2	[23]
Osteosarcoma	MG-63, HOS, Saos-2 and 143B	Increased, Decreased	Increased tumor cell necrosis rate to neoadjuvant chemotherapy; Better OS.	Facilitate proliferation, colony formation, and migration, and repress apoptosis in vitro, and tumor growth in vivo; Suppress proliferation, colony formation migration, invasion in vitro and induce apoptosis in vitro, and tumor growth in vivo	miR-618/MAEL,miR-765/APE1	[13,30,31]

Several limitations should be considered in this meta-analysis. First, all the included studies were retrospective. Second, most of studies did not directly provide HRs for survival outcomes, so we had to calculate HRs based on the Kaplan–Meier survival curves. This manual process may unavoidably cause some calculation errors. Third, there was significant heterogeneity and it may be caused by the complexity and inconsistency in many aspects of the included studies, such as follow-up time, age, gender, and therapy. Fourth, only 12 eligible studies were included into this meta-analysis, suggesting that our conclusions may be still limited by sample size. Additionally, only 1 or 2 eligible studies focused on the specific tumors, so we could not perform the pooled analysis for the specific tumors, which may largely discount the clinical significance of our meta-analysis. Last but not least, all the studies included in our meta-analysis were from China. Therefore, our conclusions could not be directly generalized to the other populations.

## Conclusion

Increased LINC00511 expression level significantly correlated with worse prognosis and higher incidence of lymph node metastasis in patients with malignant tumors, indicating that LINC00511 may be a prognostic biomarker and therapeutic target. However, our study may overestimate the clinical significance of LINC00511 expression in human malignancies due to several limitations. Therefore, further high-quality studies should be performed to further validate our findings.

## Data Availability

All data generated or analyzed during this study are included in this published article.

## Highlight

1. Role of LINC00511 in tumor

## Abbreviations

HR: hazard ratio; CI: confidence interval; OS: overall survival; LncRNA: long noncoding RNA; LINC00511: long intergenic non-protein-coding RNA 511; CCDN1: Cyclin D1; E2F1: E2F transcription factor 1; EZH2: enhancer of zeste homolog 2; LSD1: lysine-specific demethylase 1; PRC2: Polycomb repressive complex 2; CDKN1B: cyclin-dependent kinase inhibitor 1B; STXBP4: syntaxin-binding protein 4; CCDN2: cyclin D2; ZEB1: zinc finger E-box binding homeobox 1; YB1: Y-box binding protein; EYA1: eyes absent homolog 1; VEGFA: Vascular endothelial growth factor A; LAS2: large tumor suppressor, homolog 2; KLF2: Krueppel-like factor 2; CDK2/4: cyclin-dependent kinase 2/4; TAF1: TATA-box binding protein-associated factor 1; JAK2: Janus kinase 2; STAT3: activating signal transducer and activator of transcription 3; PLD1: Phospholipase D1; RXRA: retinoic X receptor alpha; MMP-2/9: matrix metalloproteinase-2/9; P-GP: P-glycoprotein; MRP1: mmultidrug resistance protein 1; LAMC2: laminin subunit gamma 2; APE1: apurinic/apyrimidinic endonuclease 1; MAEL: Maelstrom; ceRNA: competing endogenous RNA; qPCR: quantitative real-time polymerase chain reaction; TCGA: The Cancer Genome Atlas.
